# Coccidioidomycosis Seroincidence and Risk among Military Personnel, Naval Air Station Lemoore, San Joaquin Valley, California, USA^1^

**DOI:** 10.3201/eid2809.220652

**Published:** 2022-09

**Authors:** Graham C. Ellis, Charlotte A. Lanteri, Hsing-Chuan Hsieh, Paul C.F. Graf, Gabriel Pineda, Nancy F. Crum-Cianflone, Catherine M. Berjohn, Terrel Sanders, Ryan C. Maves, Robert Deiss

**Affiliations:** Explosive Ordnance Disposal Expeditionary Support Unit TWO, Virginia Beach, Virginia, USA (G.C. Ellis);; Naval Medical Center San Diego, San Diego, California, USA (G.C. Ellis, C.M. Berjohn, T. Sanders, R.C. Maves, R. Deiss);; Uniformed Services University, Bethesda, Maryland, USA (C.A. Lanteri, H.-C. Hsieh, C.M. Berjohn, R.C. Maves, R. Deiss);; The Henry M. Jackson Foundation for the Advancement of Military Medicine, Inc., Bethesda (H.-C. Hsieh, R. Deiss);; US Naval Medical Research Unit SIX, Lima, Peru (P.C.F. Graf);; US Naval Health Research Center, San Diego (P.C.F. Graf, G. Pineda);; Scripps Mercy Hospital, San Diego (N.F. Crum-Cianflone);; US Naval Medical Research Unit THREE, Accra, Ghana (T. Sanders);; Wake Forest University School of Medicine, Winston-Salem, North Carolina, USA (R.C. Maves);; University of California, San Diego (R. Deiss)

**Keywords:** coccidioidomycosis, Coccidioides, infections, endemic diseases, lung disease, fungi, respiratory infections, seroincidence, risk, military personnel, Naval Air Station Lemoore, San Joaquin Valley, California, United States

## Abstract

We conducted a retrospective cohort study that tested 2,000 US military personnel for *Coccidioides* antibodies in a disease-endemic region. The overall incidence of seroconversion was 0.5 cases/100 person-years; 12.5% of persons who seroconverted had illnesses requiring medical care. No significant association was found between demographic characteristics and seroconversion or disease.

Coccidioidomycosis is an endemic mycosis caused by inhalation of *Coccidioides immitis* or *C*. *posadasii* spores (*1*). Severe disease is infrequent, and extrapulmonary dissemination occurs in only 1% of diagnosed cases (*2**,**3*). Mild disease and varied clinical awareness of this pathogen contribute to underestimates of incidence (*4*).

The US military maintains facilities in coccidioidomycosis-endemic regions where nonimmune persons are routinely assigned. We sought to investigate the seroincidence of *Coccidioides* infection in persons stationed at a naval air station (NAS) in the San Joaquin Valley of California, USA, an area to which coccidioidomycosis is endemic (*5*). The study was approved by the Institutional Review Boards of the Uniformed Services University and Naval Health Research Center.

## The Study

We used samples collected during 2011–2017 from the Department of Defense Serum Repository (DoDSR), a program that stores serum from US service members collected during routine health screenings. We conducted a retrospective cohort study of 2,000 military service members newly transferred to NAS Lemoore, a military base in the San Joaquin Valley that employs 6,400 military personnel (*6*). Our primary objectives were to define the incidence of and risks for seroconversion at NAS Lemoore. Secondary objectives included determining the proportion of seropositive cases associated with the development of clinical disease. We queried the Armed Forces Health Services Division database to identify service members who were newly transferred to NAS Lemoore from a non-*Coccidioides*‒endemic region, as determined by postal code associated with the serum sample.

The population consisted of service members for whom the DoDSR had 1 serum sample drawn before arrival at NAS Lemoore (pretransfer) and 1 sample drawn after >12 months at NAS Lemoore (posttransfer) during 2011–2017. Posttransfer serum samples (2 mL) from eligible persons were obtained and tested for *Coccidioides* IgG and IgM by the Naval Health Research Center (San Diego, CA, USA) by using the Omega Cocci Antibody Enzyme Immunoassay (EIA) on the automated DS2 instrument (Dynex Technologies, https://www.dynextechnologies.com). Samples seropositive by EIA underwent confirmatory immunodiffusion testing at the University of California Davis Coccidioidomycosis Reference Laboratory (Davis, CA, USA). For positive or equivocal samples, we obtained pretransfer samples from the DoDSR and tested them for *Coccidioides* antibodies by EIA to determine the presence of seroreactivity (seropositive or equivocal) before the service member’s transfer to a *Coccidioides*-endemic region (Appendix).

We defined seroconversion as IgG or IgM detected in a posttransfer sample by EIA, confirmed by immunodiffusion if performed, with a seronegative pretransfer EIA result. IgG seroreactivity in a pretransfer sample was considered evidence of previous *Coccidioides* exposure. Isolated IgM seroreactivity in a pretransfer sample was considered false positive if there was IgM seroreactivity in the posttransfer sample. Cases that could not be confirmed by immunodiffusion because of testing limitations but that otherwise met the above definition were included as cases of seroconversion in our analysis.

We obtained deidentified demographic and clinical data from the Armed Forces Health Services Division. Demographics included branch of service, military rank, and occupational specialty code (Appendix). We obtained clinical diagnosis by query of the International Classification of Diseases 9th and 10th Revisions (Appendix). Case-patients who had coccidioidomycosis were considered if cases occurred while they were stationed at NAS Lemoore, within 90 days after transfer, or within 2 years if disseminated.

We calculated prevalence and incidence with 95% CI based on binomial and Poisson distributions. Prevalence was the number of positive screens divided by the number of seronaive persons on transfer. Incidence was calculated by using person-years at NAS Lemoore. We determined bivariate associations by using the Mann-Whitney U/Wilcoxon rank tests for continuous variables and the χ^2^ or Fischer exact test for categorical variables. We used a simple logistical regression model to determine predictors of seroconversion or disease and considered a 2-sided p value <0.05 statistically significant. We performed all statistical calculations by using SAS version 9.4 (https://www.sas.com).

We obtained serum and clinical data for 2,000 participants (Table 1); participants were predominately male and <27 years of age. *Coccidioides* IgG or IgM were detected (positive or equivocal) by EIA in 415 (21%) of 2,000 samples (Appendix); of those, 252 (61%) were equivocal for IgM alone and were excluded as false positives. Confirmatory testing was performed on 144 of the remaining 163 seropositive/seroequivocal samples by EIA (88.3%). Overall, 19 were positive for IgG alone, 1 positive for IgG and IgM, and 2 equivocal for IgM alone. For these 22 samples and the 19 samples that were not sent for confirmatory testing by immunodiffusion, a pretransfer sample was obtained from the DoDSR and tested for *Coccidioides* antibodies by EIA to determine previous seroreactivity. Five persons had serologic evidence of previous *Coccidioides* exposure by pretransfer EIA. Twelve participants were IgG‒/IgM+ for pretransfer and posttransfer samples and were considered false positives.

**Table 1:** Cohort demographics by seroconversion status for incidence of *Coccidioides *seroconversion among military personnel, Naval Air Station Lemoore, San Joaquin Valley, California, USA*

Variables	Seroconversion		Total	p value
	No	Yes		
Total	1,976	24	2,000	
Mean age, y (range)	23.0 (20.0–27.0)	22.5 (19.0–26.5)	23.0 (20.0–27.0)	0.5358
Sex				>0.999
M	1,657 (83.86)	20 (83.33)	1,677 (83.85)	
F	319 (16.14)	4 (16.67)	323 (16.15)	
Race/ethnicity				0.1098
Caucasian	910 (46.05)	6 (25.00)	916 (45.80)	
African American	298 (15.08)	5 (20.83)	303 (15.15)	
Asian/Pacific Islander	120 (6.07)	2 (8.33)	122 (6.10)	
American Indian/Alaskan Native	53 (2.68)	0	53 (2.65)	
Hispanic	338 (17.11)	4 (16.67)	342 (17.10)	
Other	233 (11.79)	6 (25.00)	239 (11.95)	
Unknown	24 (1.21)	1 (4.17)	25 (1.25)	
Education				0.8220
No high school	10 (0.51)	0	10 (0.50)	
High school	1,562 (79.05)	20 (83.33)	1,582 (79.10)	
Bachelor's degree or <4 y of college degree	273 (13.82)	4 (16.67)	277 (13.85)	
Master’s degree or higher	32 (1.62)	0	32 (1.60)	
Unknown	99 (5.01)	0	99 (4.95)	
Service				>0.999
Navy	1,965 (99.44)	24 (100.00)	1,989 (99.45)	
Other	11 (0.56)	0	11 (0.55)	
Grade				0.1999
Enlisted	1,791 (90.64)	24 (100.00)	1,815 (90.75)	
Warrant	4 (0.20)	0	4 (0.20)	
Officer	181 (9.16)	0	181 (9.05)	
Outdoor occupations				0.2975
Yes	1,193 (60.37)	17 (70.83)	1,210 (60.50)	
No	783 (39.63)	7 (29.17)	790 (39.50)	

*Values are no. (%) unless indicated otherwise.

A total of 24 (1.2%) participants met our definition for seroconversion and were included in our analysis (Table 1). Of those, 20 (83.3%) had positive immunodiffusion results by confirmatory testing. Four (16.7%) showed evidence of seroconversion on posttransfer EIAs (one IgG+/IgM‒, 1 IgG/IgM equivocal, and 2 IgG equivocal/IgM‒) and negative pretransfer EIA results but lacked sufficient posttransfer sample volume for confirmation; they were included as seroconversion cases.

Annual incidence ranged from 0 to 1.32 cases/100 person-years; overall seroconversion incidence was 0.5 cases/100 person-years (Figure; Appendix). Three (12.5%) of the 24 newly seropositive persons were given a diagnosis of coccidioidomycosis or pneumonia after seroconversion. No disseminated infections were diagnosed. No disease was documented in persons who had *Coccidioides* antibodies before arrival. Two coccidioidomycosis diagnoses were for seronegative persons.

**Figure:**
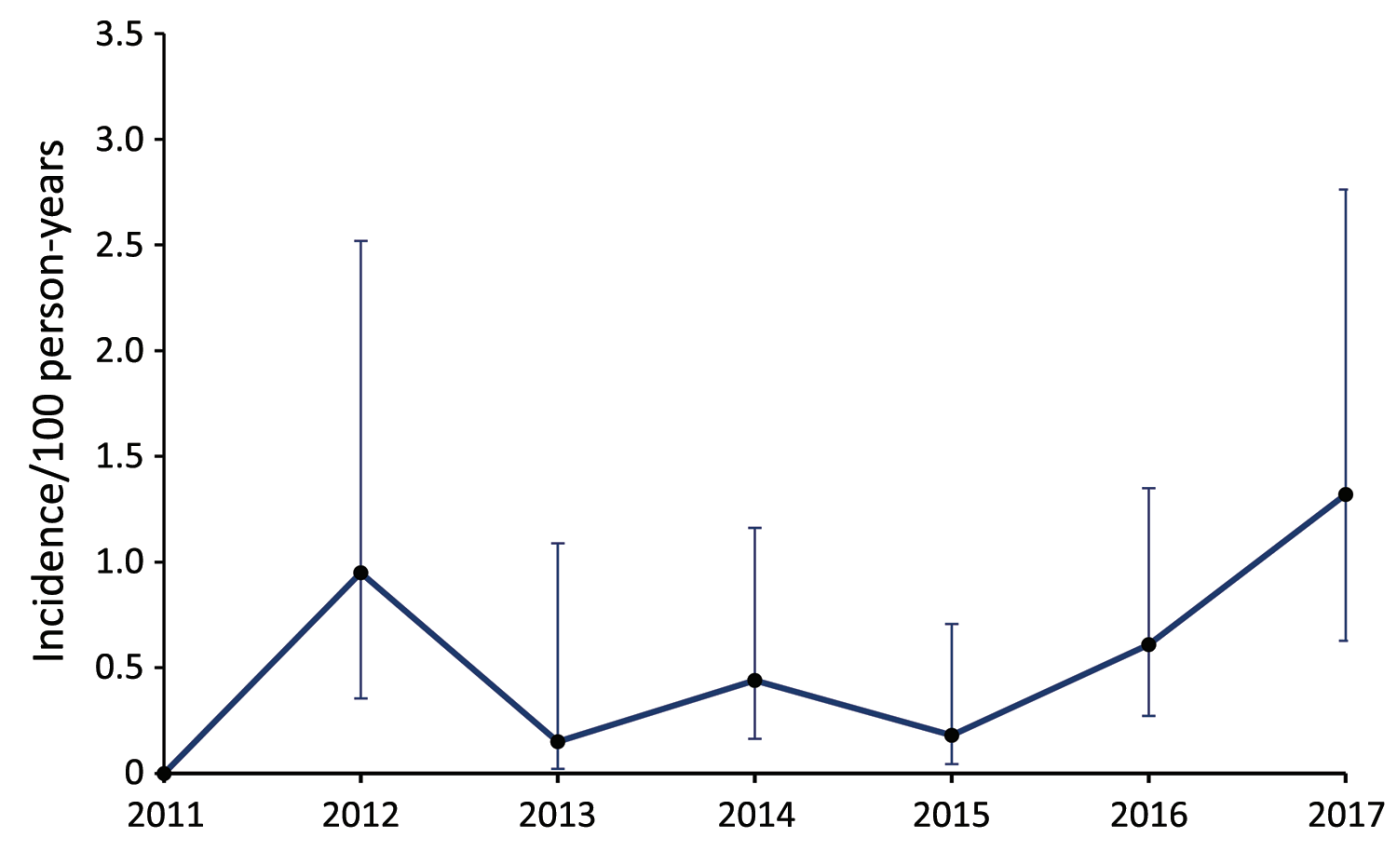
Incidence of *Coccidioides *seroconversion among military personnel, Naval Air Station Lemoore, San Joaquin Valley, California, USA. Error bars indicate 95% CIs.

We found by bivariate and regression analyses no statistically significant associations between any demographic variable and seropositivity or disease (Tables 1,2). We did not observe statistically significant differences in seropositivity between racial/ethnic groups or by occupation. Seropositivity was significantly associated with disease (p = 0.027) (Table 2).

**Table 2:** Cohort demographics by coccidioidomycosis-like diagnosis for incidence of *Coccidioides *seroconversion among military personnel, Naval Air Station Lemoore, San Joaquin Valley, California, USA*

Variables	Diagnosis		Total	p value
	No	Yes		
Seroconversion				0.0267
Yes†	27 (1.38)	3 (6.82)	30 (1.50)	
No	1,929 (98.62)	41 (93.18)	1,970 (98.50)	
Mean age, y (range)	24.0 (20.0–27.0)	21.0 (19.5–25.0)	23.0 (20.0–27.0)	0.0572
Sex				0.1067
M	1,644 (84.05)	33 (75.00)	1,677 (83.85)	
F	312 (15.95)	11 (25.00)	323 (16.15)	
Race/ethnicity				0.5885
Caucasian	892 (45.60)	24 (54.55)	916 (45.80)	
African American	299 (15.29)	4 (9.09)	303 (15.15)	
Asian/Pacific Islander	118 (6.03)	4 (9.09)	122 (6.10)	
Hispanic	335 (17.13)	7 (15.91)	342 (17.10)	
Other	287 (14.67)	5 (11.36)	292 (14.60)	
Unknown	25 (1.28)	0	25 (1.25)	
Education				>0.999
No high school	10 (0.51)	0	10 (0.50)	
High school	1,545 (78.99)	37 (84.09)	1,582 (79.10)	
Bachelor’s degree or less	271 (13.85)	6 (13.64)	277 (13.85)	
Master's degree or higher	32 (1.64)	0	32 (1.60)	
Unknown	98 (5.01)	1 (2.27)	99 (4.95)	
Service				>0.999
Navy	1,945 (99.44)	44 (100.00)	1,989 (99.45)	
Other	11 (0.56)	0	11 (0.55)	
Grade				0.1799
Enlisted	1,772 (90.59)	43 (97.73)	1,815 (90.75)	
Warrant/officer	184 (9.41)	1 (2.27)	185 (9.25)	
Outdoor occupation	1,186 (60.63)	20 (45.45)	1,210 (60.50)	0.4139
Total	1,956	44	2,000	

*Values are no. (%) unless indicated otherwise. †Includes previous seroconversion.

## Conclusions

Our observed incidence of 0.5 cases/100 person-years is lower than published observations of asymptomatic infection (*7**–**12*). We found no statistically significant association between seropositivity and any demographic variable but were limited by low rates of seroconversion and disease. Prospective *Coccidioides* skin testing at 4 military airfields in southern California, including Lemoore, during 1941–1945 found annual conversion rates as high as 12.43%, decreasing to 1.43% and 2.86% in the 2 years after environmental controls were put in place (*7*). Our incidence rates appear lower than those previously reported and might better represent seroconversion in persons with average dust exposure in the modern era.

The first limitation of our study is that performance of serologic analysis for *Coccidioides* infection depends on time from exposure and varies by method (*13*), which might explain the discordance between coccidioidomycosis diagnoses identified in service members who showed negative test results. High rates of seroreactivity and funding constraints complicated serologic definitions that were limited by discordance between EIA and immunodiffusion results. However, we confirmed seronegativity by immunodiffusion in more than half of the 252 posttransfer IgG/IgM-equivocal samples, instilling confidence in classifying these results as negatives. Our sample size and cohort homogeneity limited our ability to detect significant risk factors for infection. The retrospective nature of our study could miss mild disease cases. Furthermore, military personnel are often healthy and have few underlying illnesses, potentially explaining the low rate of symptomatic illness in the cohort.

In summary, we found that coccidioidomycosis was uncommon in a military population newly transferred to a disease-endemic region, and progression to clinically apparent disease was infrequent. Longitudinal prospective studies are needed to monitor epidemiologic trends over time and to determine disease risks in diverse populations. Although these low rates of seroincidence and disease are reassuring, caution is warranted when considering this pathogen with complex disease ecology.

Appendix Additional information on coccidioidomycosis seroincidence and risk among military personnel, Naval Air Station Lemoore, San Joaquin Valley, California, USA.
