# Supplementary material for: Coccidioidomycosis Seroincidence and Risk among Military Personnel, Naval Air Station Lemoore, San Joaquin Valley, California, USA
Source: Emerg Infect Dis. 2022 Sep;28(9):1842–6. doi: 10.3201/eid2809.220652 (PMC9423930; doi:10.3201/eid2809.220652)
Supplement: Appendix — Additional information on coccidioidomycosis seroincidence and risk among military personnel, Naval Air Station Lemoore, San Joaquin Valley, California, USA. [file 22-0652-Techapp-s1.pdf]

Article DOI: https://doi.org/10.3201/eid2809.220652

Coccidioidomycosis Seroincidence and Risk Among Military Personnel, Naval Air Station Lemoore, San Joaquin Valley, California, USA

Appendix

Appendix Table 1. Distribution of DOD Occupational Specialty Codes and Outdoor Classification

DoD Specialty Code	Description	Frequency, no. (%)
Indoor Occupations		790 (39.5)
104100	Artillery and Gunnery	2 (0.10)
105000	Air Crew, General	1 (0.05)
106000	Boatswains	1 (0.05)
106100	Navigators	1 (0.05)
106300	Seamanship, General	1 (0.05)
110000	Radio/Radar, General	1 (0.05)
110200	Navigation, Communication, and Countermeasure, N.E.C.	270 (13.5)
112100	Missile Guidance and Control	2 (0.10)
115000	ADP Computers, General	9 (0.45)
119800	Electronic Instruments, N.E.C.	2 (0.10)
122100	Radar	4 (0.20)
122200	Air Traffic Control	63 (3.15)
124200	Image Interpretation	13 (0.65)
130000	Medical Care and Treatment, General	40 (2.00)
142000	Weather, General	3 (0.15)
143300	Divers	3 (0.15)
150100	Recruiting and Counseling	3 (0.15)
151000	Administration, General	26 (1.30)
151200	Legal	3 (0.15)
152000	Combined Personnel and Administration, General	12 (0.60)
152100	First Sergeants, Sergeants Major, and Leading Chiefs	5 (0.25)
155100	Supply Administration	93 (4.65)
156100	Chaplain's Assistants	4 (0.20)
162300	Interior Communications	4 (0.20)
165100	Main Propulsion	2 (0.10)
165200	Auxiliaries	5 (0.25)
166200	Electric Power	1 (0.05)
170200	Machinists	2 (0.10)
180000	Food Service, General	32 (1.60)
181100	Motor Vehicle Operators	1 (0.05)
182300	Sales Store	6 (0.30)
183000	Law Enforcement, General	5 (0.25)
192000	Undesignated Occupations, General	1 (0.05)
195000	Not Occupationally Qualified, General	1 (0.05)
220100	Fixed-Wing Fighter/Bomber Pilots	22 (1.10)
220200	Other Fixed-Wing Pilots	21 (1.05)
220400	Aircraft Crews	58 (2.90)
220700	Operations Staff	1 (0.05)
230100	General Intelligence	5 (0.25)
240600	Missile Maintenance	1 (0.05)
250600	Legal	6 (0.30)
250700	Chaplains	3 (0.15)
260100	Medical Corps	18 (0.90)
260300	Dental Corps	6 (0.30)
260500	Nurse Corps general	6 (0.30)
260700	Veterinarians	1 (0.05)

DoD Specialty Code	Description	Frequency, no. (%)
260900	Health Services Administration Officers	11 (0.55)
270100	General Administrators	4 (0.20)
280200	Supply	4 (0.20)
290200	Students	1 (0.05)
Outdoor Occupations		1,210 (60.5)
155500	Aviation Maintenance Records and Reports	95 (4.75)
160000	Aircraft, General	16 (0.80)
160100	Aircraft Engines	263 (13.15)
160200	Aircraft Accessories	197 (9.85)
160300	Aircraft Structures	277 (13.85)
160400	Aircraft Launch Equipment	53 (2.65)
164600	Aviation Ordnance	212 (10.6)
171000	Construction, General	5 (0.25)
171100	Steel working	4 (0.20)
171300	Construction Equipment Operation	4 (0.20)
179000	Other Crafts workers, N.E.C., General	4 (0.20)
186000	Forward Area Equipment Support, General	63 (3.15)
220500	Ground and Naval Arms	3 (0.15)
240400	Aviation Maintenance and Allied	14 (0.70)

Appendix Table 2. International Statistical Classification of Diseases and Related Health Problems (ICD) Codes considered markers of coccidioidomycosis

ICD-9 code	Diagnosis
483	Pneumonia due to other specified organism
485	Bronchopneumonia, organism unspecified
486	Pneumonia, organism unspecified
513	Abscess of lung and mediastinum
510	Empyema
511.X	Pleurisy and pleural effusion
519	Other diseases of respiratory system
518.5	Acute respiratory distress syndrome
518.81	Respiratory failure, acute
730	Osteomyelitis
114	Coccidioidomycosis
ICD-10 Code	Diagnosis
J16	Pneumonia due to other infectious organisms
J17	Pneumonia in disease classified elsewhere
J18	Pneumonia, unspecified organism
J22	Unspecified acute lower respiratory infection
J85	Abscess of lung and mediastinum
J86	Pyothorax
J90	Pleural effusion
J91	Pleural effusion in conditions classified elsewhere
J94	Other pleural conditions
J96	Respiratory failure
J99	Respiratory disorders in disease classified elsewhere
M86	Osteomyelitis
B38	Coccidioidomycosis
B38.0	Acute pulmonary coccidioidomycosis
B38.1	Chronic pulmonary coccidioidomycosis
B38.2	Pulmonary coccidioidomycosis, unspecified
B38.3	Cutaneous coccidioidomycosis
B38.4	Coccidioidomycosis meningitis
B38.7	Disseminated coccidioidomycosis
B38.8	Other forms of coccidioidomycosis

Appendix Table 3. Annual rates of seroconversion

Year	No. cases*	Person-years	Incidence rate†	95% CI
Overall	24	4,807.25	0.50	(0.33–0.74)
2011	0	162.96	0.00	–
2012	4	423.04	0.95	(0.35–2.52)
2013	1	652.22	0.15	(0.02–1.09)
2014	4	917.31	0.44	(0.16–1.16)
2015	2	1,130.57	0.18	(0.04–0.71)
2016	6	989.60	0.61	(0.27–1.35)
2017	7	531.55	1.32	(0.63–2.76)

*Includes possible seroconversion cases.
†Per 100 person-years.

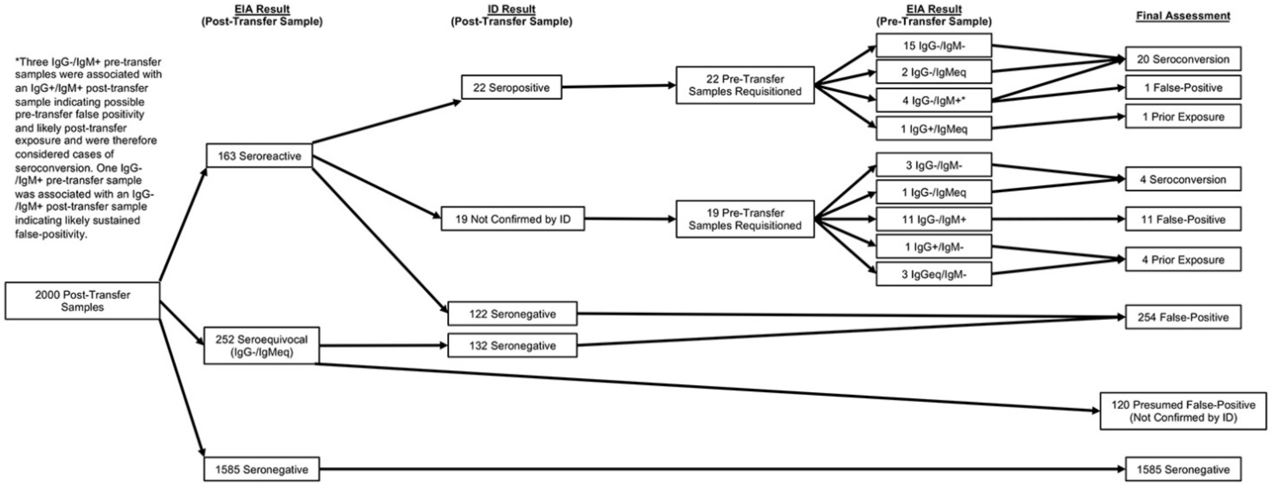

Appendix Figure. Sample testing flow diagram. EIA, enzyme immunoassay; ID, immunodiffusion.
